# One-year efficacy of a lifestyle behavioural intervention on physical and mental health in people with severe mental disorders: results from a randomized controlled trial

**DOI:** 10.1007/s00406-023-01684-w

**Published:** 2023-09-04

**Authors:** M. Luciano, G. Sampogna, E. D’Ambrosio, A. Rampino, M. Amore, P. Calcagno, A. Rossi, R. Rossi, C. Carmassi, L. Dell’Osso, E. Bianciardi, A. Siracusano, Bianca Della Rocca, M. Di Vincenzo, LIFESTYLE Working Group, Valeria Del Vecchio, Claudio Malangone, Emiliana Mancuso, Claudia Toni, Antonio Volpicelli, Ileana Andriola, Pierluigi Selvaggi, Martino Belvederi Murri, Ramona Di Stefano, Francesca Pacitti, Valerio Dell’Oste, Sara Fantasia, Virginia Pedrinelli, Giorgio Di Lorenzo, Cinzia Niolu, A. Fiorillo

**Affiliations:** 1 Department of Psychiatry, University of Campania “L. Vanvitelli”, Largo Madonna Delle Grazie 80039, Naples, Italy; 2 Department of Basic Medical Science, Neuroscience and Sense Organs, University of Bari “Aldo Moro”, Bari, Italy; 3 Section of Psychiatry, Department of Neuroscience, Ophthalmology, Genetics and Infant-Maternal Science, University of Genoa, Genoa, Italy; 4 Department of Biotechnological and Applied Clinical Sciences, University of L’Aquila, L’Aquila, Italy; 5 Department of System Medicine, University of Rome Tor Vergata, Rome, Italy; 6 Department of Clinical and Experimental Medicine, University of Pisa, Pisa, Italy

**Keywords:** LIFESTYLE, Comorbidity, Schizophrenia, Bipolar disorder, Depression, RCT, HOMA-IR index, Framingham Risk score, BMI, Waist circumference

## Abstract

This multicentric randomized controlled trial (RCT), carried out in six Italian University mental health sites, aims to test the efficacy of a six-month psychosocial intervention (LYFESTYLE) on Body Mass Index (BMI), body weight, waist circumference, fasting glucose, triglycerides, cholesterol, Framingham and HOmeostasis Model Assessment of insulin resistance (HOMA-IR) indexes in patients with schizophrenia, bipolar disorder, and major depression. Moreover, the efficacy of the intervention has also been tested on several other physical and mental health domains. Patients were randomly allocated to receive the six-month experimental intervention (LIFESTYLE) or a behavioural control intervention. All enrolled patients were assessed at baseline and after one year. We recruited 401 patients (206 in the experimental and 195 in the control group) with a diagnosis of schizophrenia or other psychotic disorder (29.9%), bipolar disorder (43.3%), or major depression (26.9%). At one year, patients receiving the experimental intervention reported an improvement in body mass index, body weight, waist circumference, HOMA-IR index, anxiety and depressive symptoms and in quality of life. Our findings confirm the efficacy of the LIFESTYLE intervention in improving physical and mental health-related outcomes in patients with severe mental illnesses after one year.

## Introduction

Patients with severe mental disorders (SMI; namely schizophrenia and other psychotic disorders; bipolar disorders; and major depression) have a significantly lower life expectancy compared to the general population, largely as a consequence of the increased prevalence of cardiovascular and metabolic diseases, including obesity, dyslipidaemia, diabetes mellitus, and metabolic syndrome [1]. The high rates of comorbidity between mental and physical illnesses is mainly due to the adoption of unhealthy lifestyle behaviours (i.e., poor dietary habits and physical activity, heavy smoking, alcohol or drug abuse) and reduced access to screening programmes and check-up visits for physical disorders. Other factors include the erroneous attribution by physicians of patients’ somatic complaints to mental rather than to physical diseases [2, 3] and the adverse events of several psychotropic medications, including antipsychotic medications, mood stabilizers and antidepressants [4].

Because of the high rates of physical comorbidities and premature mortality in patients with severe mental disorders [5], several lifestyle interventions have been recently developed, with the aim to improve their cardiovascular and metabolic parameters [6]. These interventions can be defined as structured approaches that help individuals to perform physical activities, stop smoking, manage body weight, have a balanced and healthy diet and engage in healthy programmes [7]. Some of these lifestyle interventions effectively reduce Body Mass Index (BMI), body weight [8, 9], triglyceride levels and fasting glucose [10]. BMI reductions seem greater and more persistent after three, six and 12 months [11]. Some of these approaches also reduce waist circumference, which is considered a more reliable cardiovascular risk factor than BMI [12], although its assessment is hampered by the lack of reliable standardized assessment tools [11].

The efficacy of lifestyle interventions has also been tested in terms of analytical blood parameters (i.e., blood levels of total cholesterol, triglycerides, insulin and fasting glucose), despite heterogeneous results do not allow to draw firm conclusions. According to systematic reviews [10–13], analytical blood parameters significantly decrease after the provision of lifestyle behavioural interventions focusing on diet and physical activity. However, most of these studies have included short follow-ups (i.e., up to three months), while longer follow-ups are rarely available. Of note, no study has specifically investigated the impact of lifestyle interventions on complex cardiovascular indexes, such as the HOmeostasis Model Assessment of insulin resistance (HOMA- IR) and the Framingham indexes, which are more reliable predictors of cardiometabolic disorders [14].

Lifestyle interventions also have a positive impact on patients’ psychosocial well-being [15–17], quality of life and severity of symptoms [18, 19], such as psychotic [20–22], depressive [22, 23] and anxiety [24] symptoms.

However, most randomized controlled trials (RCTs) carried out so far present several methodological weaknesses, with up to 64% of studies rated as at high risk of bias [8]. The most frequently reported biases include the process of randomization, lack of blinding assessments, missing outcome data, imprecise outcome reporting [8], small sample sizes, statistical heterogeneity and lack of active control groups [3].

The LIFESTYLE trial has been designed to assess the efficacy of a psychosocial group intervention promoting healthy lifestyle behaviours on patients’ physical health, compared to a brief psychoeducational group intervention. In particular, the efficacy of the experimental intervention has been tested on the reduction of Body Mass Index (BMI), the improvement of anthropometric and haematological parameters, and reduction of cardiovascular risk and insulin resistance. Other secondary outcomes include the improvement of patients’ eating habits (e.g., reduction in fat food and increase in fruits and vegetables), smoking habits (i.e., number of cigarettes smoked per day), sleeping habits (i.e., number of hours slept per night), physical activity (i.e., increase in walking time every day), personal and social functioning, presence of physical illnesses, and adherence to medications. The overall study aims, with the relevant assessment instruments, are reported in Table 1.

**Table 1:** Global overview of assessment instruments

Assessment tools	Covered domain
*Lifestyle behaviours*	
Short food frequency questionnaire	Diet
International physical activity questionnaire	Physical activity
Fagerström test for nicotine	Nicotine addiction
Questionnaire on sexual health	Sexual habits
Pittsburgh sleep quality index	Sleep quality
Leeds dependence questionnaire	Drug dependence
Morinsky medication adherence scale	Adherence to pharmacological medicine
*Psychiatric and psychosocial assessment*	
The structured clinical interview for DSM-5 (SCID-5)	Psychiatric diagnosis
Brief psychiatric rating scale	Psychopathological status
Manchester short assessment of quality of life	Quality of life
Recovery style questionnaire	Styles of recovery
Personal and social performance scale	Psychosocial functioning
MATRIC consensus trial making test – Part A, BACS symbol coding, Category Fluency-Animal Naming	Cognitive functioning
Internalized stigma of mental illness (ISMI)	Internalized stigma
*Cardio-metabolic risk assessment and anthropometric parameters*	
HOMA-IR index	Insulin resistance
Framingham index	Cardiovascular risk
Cumulative illness rating scale	Physical comorbidities
Anthropometric schedule	Weight, height, BMI, waist circumference, blood pressure, resting heart rate, HDL, LDL, and overall level of cholesterol, blood glucose, triglycerides, and blood insulin

In this paper, we report data on the efficacy at one year of the LIFESTYLE intervention on BMI, fasting glucose, blood levels of triglycerides, cholesterol, Framingham and HOMA-IR indexes, weight, waist circumference, and subscales of the Cumulative Illness Rating Scale (CIRS). Moreover, we also report data on the impact of the experimental intervention on mental health domains, such as severity of psychiatric symptoms and quality of life.

## Methods

The study was carried out at the outpatient units of six Italian sites (University of Campania “L. Vanvitelli in Naples, University of Bari, L’Aquila Genova, Pisa and Rome Tor Vergata). Eligible patients were randomized by the Naples coordinating centre taking into account center, age, gender, and educational level, with a 1:1 ratio.

The following inclusion criteria were considered: (1) age between 18 and 65 years; (2) a diagnosis of schizophrenia and other primary psychosis, unipolar depression, or bipolar disorder according to the DSM-5 and confirmed by the Structured Clinical Interview for DSM-5 (SCID-5) [25]; (3) a BMI ≥ 25; (4) at least one monthly contact with the referring psychiatric unit for the three months before enrollment. Patients who were not able to perform moderate physical activity as well as pregnant or breastfeeding women were excluded. Patients with intellectual disability or severe cognitive impairment were considered not eligible, as well as patients who were experiencing a clinically relevant worsening of psychiatric symptoms (i.e., requiring a substantial change in the therapeutic dosage of psychiatric medications or an access to emergency care or hospitalization) in the previous three months.

For each participating center, three mental health professionals (at least one of them being a psychiatrist) participated in a five-day training course on the main characteristics of the two interventions. Supervision meetings have been regularly provided to mental health professionals by phone, by e-mail or in presence during the whole duration of the intervention. Researchers involved in patients’ assessments were blinded with respect to patient’s allocation. The study was carried out in accordance with the Declaration of Helsinki and with local regulations. A formal ethical approval for conducting the trial was obtained by the Coordinating Center’s Ethics Committee, which approved the study protocol in January 2017 (approval number: prot. 64), and by the Ethics Committee of each participating centre.

## Interventions

### The LIFESTYLE intervention

The LIFESTYLE intervention is a five-month, multicomponent psychosocial intervention provided to groups of five to ten patients every 7–10 days, covering the following lifestyle topics: (1) healthy diet; (2) physical activity; (3) smoking habits; (4) adherence to medications; (5) adoption of risky behaviors; (6) promotion of circadian rhythms. Each topic is divided in the following components: (a) information on the risks of unhealthy lifestyle behaviours and on benefits of healthy lifestyle; (b) motivational interview and problem-solving session on changing the unhealthy lifestyle behaviors; (c) identification of personal healthy lifestyle goals for each participant. All sessions are organized in order to stimulate discussion, small workgroups and active interaction among participants. At the end of each meeting, a 20-min group session of moderate physical activity is scheduled. The intervention has been developed according to the guidelines on the management of physical health in people with mental disorders of the World Health Organization [26, 27], the European Association for the Study of Diabetes [28], the European Society of Cardiology [29], and the European Psychiatric Association [30].

The educational material has been developed according to the following phases: (1) analysis of the scientific literature; (2) evaluation of handbooks and manuals on other psychosocial lifestyle behavioural interventions; (3) focus groups with expert researchers and clinicians, and with users and carers, in order to identify the most relevant needs to be addressed; (4) development of an ad-hoc manual for mental health professionals with a detailed description of the whole intervention. Leaflets and other written materials are given to patients, whenever relevant.

Key features of the intervention are the inclusion of the motivational interview and the identification of one or two personal healthy lifestyle goals. After the identification of personal goals, professionals motivate patients to change their lifestyle and teach them problem-solving strategies for sustaining the behavioral change [31].

### Control intervention

The control intervention consists of 5 psychoeducational sessions, provided every 7 days to groups of 5–10 patients, on: (1) promotion of healthy diet and physical activity; (2) detection of early warning signs; (3) pharmacological treatments for severe mental disorders and their side effects; (4) stress management; (5) problem-solving skills. Moreover, the intervention includes an introductory session, in which professionals explain aims, format, duration and timing of the intervention. Educational materials and leaflets are provided to participants during and at the end of each session.

### Assessment procedures

Patients were assessed at baseline and after six, 12 and 24 months. Twelve months’ follow-up data (T2) were analysed for the purposes of this paper. Results at six months’ evaluation (T1) can be found in Luciano et al. [4, 32].

Patients’ mental health status and psychosocial functioning were assessed through: (a) the Brief Psychiatric Rating Scale (BPRS) [34]; (b) the Personal and Social Performance Scale (PSP) [35]; (c) the 17-item Manchester Short Assessment of Quality of Life (MANSA) questionnaire [36]; (d) the MATRICS Consensus Trail Making Test – part A, Brief Assessment of Cognition in Schizophrenia: Symbol Coding, and the Category Fluency-Animal Naming [37, 38]; e) the Pattern of Care Schedule (PCS), modified version [33]. The BPRS subscales were calculated according to Shafer et al. [39] in “Positive Symptoms”, “Negative Symptoms”, “Affectivity”, “Resistance”, and “Activation”.

Patients’ physical health was assessed through: (a) the Cumulative Illness Rating Scale (CIRS) [40]; (b) weight, height, Body Mass Index (BMI), waist circumference, blood pressure, resting heart rate, overall blood levels of cholesterolemia, low-density lipoprotein (LDL) cholesterolemia, high-density lipoprotein (LDL) cholesterolemia, blood glucose, serum triglycerides; (c) the homeostasis model assessment of insulin resistance (HOMA-IR) [41].

Cohen’s Kappa coefficient was satisfactory for PSP total score (K value = 0.918) and BPRS (K value ranging from 0.835 to 0.972). A 100% agreement rate was found for the SCID-5 diagnoses.

### Statistical analyses

Statistical analyses were conducted according to the “Intention to Treat” principle. Missing data were handled using the Last Observation Carried Forward (LOCF). Descriptive statistics (frequency table, means and SD) were calculated for the experimental and control groups at baseline and at the end of the intervention. In each group (experimental vs. control), Student *t*-test for paired sample was used to test differences between baseline and 12-month follow-up with respect to outcome variables in the total sample and within diagnostic subgroups. Differences in socio-demographic and clinical characteristics among completers and drop-outs were analyzed with Student t-test for paired sample or χ^2^, as appropriate. Corrections for multiple comparisons were performed and Bonferroni-corrected p values were provided.

Generalized estimating equation models (GEE) were performed in order to assess the efficacy of the LIFESTYLE intervention on primary and secondary outcomes. Those mental and physical-related domains which were significantly improved in the experimental intervention were included as dependent variables. Covariates included cognitive functioning, age, gender, years of education, duration of illness and psychosocial functioning. Covariates were selected among those identified by the literature as at higher impact on the efficacy of behavioral interventions. GEE models were adjusted according to pharmacological treatment and diagnosis, which were included in the models as dummy variables.

## Results

401 patients were recruited; 206 of them were allocated in the experimental intervention and 195 in the control group. 173 participants completed the intervention, 87 from the experimental group and 86 from the control group. Main reasons for drop-out were: logistic difficulties to reach the unit to attend the sessions (27%); not being anymore in charge to the mental health unit (30%); worsening of psychiatric symptoms (20%); lack of interest (18%); refusal to complete the twelve-month follow-up assessments (5%). Baseline socio-demographic and clinical characteristics did not differ between completers and drop-outs in both groups.

Of the 401 recruited patients, 57% were female, with a main age of 45.8 ± 11.8 and with a main diagnosis of bipolar disorder (43.3%), psychotic disorder (29.6%) and major depression (27.1%). They were in charge to the local mental health service since 5.9 ± 6.9 years; all of them were receiving at least one psychotropic drug: 35% were given one pharmacological agent, 39% two psychotropic medications, 21% three, and 5% of them were treated with four or more pychotropics.

Mean body weight was 91.4 ± 17.4 kg; BMI was 32.5 ± 5.5, and waist circumference was 109.3 ± 14.2 cm. Patients’ socio-demographic, clinical and metabolic characteristics did not differ between the two groups (Table 2).

**Table 2:** Socio-demographic and clinical characteristics of the sample

	Experimental group (N = 206)	Control group (N = 195)
Gender, female, % (*N*)	55.3 (114)	59 (115)
Age, M (sd)	45.9 (11.6)	45.3 (12.1)
With partner, yes % (*N*)	47.1 (97)	48.2 (94)
Education (years), M (sd)	11.7 (2.6)	11.7 (3.1)
Employed, yes, % (*N*)	37.6 (77)	33.8 (66)
*Diagnosis*, % (*N*)		
Bipolar disorder	43.2 (89)	43.6 (85)
Major depression	24.8 (51)	29.2 (57)
Psychotic disorder	32.0 (66)	27.2 (53)
*BMI*, M (sd)		
Male	31.9 (4.3)	31.8 (4.6)
Female	32.4 (5.9)	33.6 (6.3)
*Waist circumference*, M (sd)		
Male	113.4 (12.4)	111.0 (11.7)
Female	104.9 (15.0)	109.1 (15.2)
Months in charge to the mental health service, M (sd)	68.7 (81.5)	74.5 (84.0)
Duration of illness, M (sd)	16.2 (11.7)	16.4 (22.4)
BPRS negative symptoms subscale, M (sd)	7.7 (3.1)	7.6 (3.1)
BPRS positive symptoms subscale, M (sd)	5.3 (2.0)	5.5 (2.1)
BPRS affectivity subscale, M (sd)	8.7 (3.0)	8.9 (3.2)
BPRS activity subscale, M (sd)	4.7 (1.9)	4.8 (1.8)
BPRS resistance subscale, M (sd)	3.9 (4.1)	4.1 (1.8)
B-MCCB, BACS Symbol coding, M (sd)	34.5 (14.2)	34.5 (13.5)
B-MCCB, animal naming, M (sd)	18.2 (5.7)	17.5 (5.1)
B-MCCB, trial making test A, M (sd)	52.8 (30.5)	51.9 (26.6)
Personal and social performance, total score, M (sd)	66.5 (14.8)	69.6 (18.7)
MANSA, total score, M (sd)	4.0 (1.0)	4.2 (1.0)

*MANSA* Manchester Short Assessment of Quality of Life, *B-MCCB* Brief MATRICS Consensus Cognitive Battery, *PSP* Personal and Social Performance Scale, *BPRS* Brief Psychiatric Rating Scale, *BACS* Brief Assessment of Cognition in Schizophrenia, *M* Mean, *sd* standard deviation

Efficacy of the LIFESTYLE intervention at 1 one-year follow-up.

At one year, we observed a reduction in BMI (from 32.2 ± 5.2 at T0 to 30.9 ± 5.2 at T2, p < 0.01), body weight (from 91.8 ± 17.2 to 87.6 ± 16.9, p < 0.01), waist circumference (from 108.6 ± 14.4 to 104.2 ± 13.7, p < 0.01), CIRS comorbidity index (from 0.3 ± 1.6 to 0.04 ± 0.2, p < 0.01) and HOMA-IR index (from 4.3 ± 5.5 to 3.1 ± 2.9, p < 0.01) in patients receiving the LIFESTYLE intervention. Moreover, a reduction in the levels of serum triglycerides (from 162.5 ± 78.1 mg/dL at T0 to 131.4 ± 76.0 mg/dL; p < 0.001 at T2), and an improvement of high-density lipoprotein cholesterolemia (from 46.2 ± 14.6 mg/dL to 50.9 ± 26.7 mg/dL; p < 0.05) were found in treated patients at T2 follow-up (Table 3).

**Table 3:** Comparisons of physical health-related domains between the two groups

	Experimental treatment		Control group	
	Baseline (*N* = 206)	One-year follow-up (*N* = 87)	Baseline (*N* = 195)	One-year follow-up (*N* = 86)
BMI, kg/m^2^, M (sd)	32.2 (5.2)	30.1 (5.2)**	32.9 (5.8)	32.3 (6.1)
Body weight, M (sd)	91.8 (17.2)	87.6 (16.9)**	92.1 (17.6)	90.3 (17.0)
Waist circumference, M (sd)	108.6 (14.4)	104.2 (13.4)**	109.9 (13.7)	107.7 (16.2)
CIRS, severity index, M (sd)	0.3 (0.3)	0.03 (0.2)	0.3 (0.3)	0.3 (0.3)
CIRS, comorbidity index, M (sd)	0.3 (1.6)	0.0 (0.2)**	0.3 (1.2)	0.2 (0.5)
Systolic blood pressure, M (sd)	125.6 (13.6)	124.0 (12.4)	125.6 (13.5)	124.5 (14.1)
Diastolic blood pressure, M (sd)	81.1 (9.3)	80.0 (7.9)	80.3 (8.6)	80.0 (10.1)
Blood glucose, mg/dL, M (sd)	95.3 (20.9)	92.6 (24.7)	95.6 (32.3)	95.4 (33.3)
Total cholesterolemia, mg/dL, M (sd)	192.7 (42.0)	183.1 (44.3)	186.9 (39.6)	181.9 (40.9)
Total LDL cholesterolemia, mg/dL, M (sd)	120.9 (36.1)	119.1 (78.8)	117.4 (33.7)	115.0 (34.5)
Total HDL cholesterolemia, mg/dL, M (sd)	46.1 (14.6)	50.9 (26.7)*	45.9 (14.7)	46.3 (16.9)
Serum triglycerides, mg/dL, M (sd)	162.5 (78.1)	131.4 (75.9)***	161.0 (67.7)	159.3 (60.1)
Framingham risk score, total score, M (sd)	9.8 (8.1)	9.1 (9.5)	8.9 (6.8)	8.0 (6.4)
HOMA-IR-IR index, M (sd)	4.3 (5.6)	3.1 (2.9)**	4.7 (5.9)	5.4 (12.2)

*BMI* Body Mass Index, *CIRS* Cumulative Illness Rating Scale, *HOMA-IR-IR* Homeostatic Model Assessment of Insulin Resistance, *LDL* Low-density lipoprotein, *HDL* High-density lipoprotein, *M* Mean, *sd* standard deviation

*p < 0.05; **p < 0.01; ***p < 0.001

Corrected p-value for multiple comparisons: *p < 0.01; ** p < 0.003; ***p < 0.00025

A reduction in the “Affectivity” (from 8.7 ± 3.0 to 7.2 ± 2.5, p < 0.001), “Activity” (from 4.7 ± 1.9 to 4.2 ± 1.3, p < 0.01) and “Negative Symptoms” (from 7.7 ± 3.1 to 7.0 ± 2.7, p < 0.05) subscales of BPRS were reported, as well as a significant improvement in perceived quality of life (MANSA total score from 4.0 ± 1.0 to 5.3 ± 0.8, p < 0.01) were found in the group receiving the LIFESTYLE intervention (Table 4). There were no statistical changes in the control group with respect to physical and mental health-related outcomes.

**Table 4:** Comparisons of mental health-related domains in the two groups

	Experimental treatment		Control group	
	Baseline (*N* = 206)	One-year follow-up (*N* = 87)	Baseline (*N* = 195)	One-year follow-up (*N* = 86)
BPRS affectivity subscale, M (sd)	8.7 (3.0)	7.2 (2.5)***	8.9 (3.2)	8.9 (2.5)
BRPS negative symptoms subscale, M (sd)	7.7 (3.1)	7.0 (2.7)*	7.6 (3.1)	7.3 (2.6)
BPRS, positive symptoms subscale, M (sd)	5.3 (2.0)	5.2 (1.9)	5.5 (2.1)	5.4 (2.0)
BPRS, activity subscale, M (sd)	4.7 (1.9)	4.2 (1.3)**	4.8 (1.8)	4.6 (1.6)
BPRS, resistance subscale, M (sd)	3.9 (4.1)	4.1 (2.1)	4.1 (1.8)	4.1 (2.0)
MANSA total score, M (sd)	4.0 (1.0)	5.3 (0.8)**	4.18 (1.0)	4.3 (1.1)
PSP total score, M (sd)	66.5 (14.8)	66.9 (16.2)	69.6 (18.7)	67.7 (15.5)
B-MCCB, symbol coding	34.5 (14.0)	35.4 (14.2)	34.5 (13.4)	34.8 (12.)
B-MCCB, category fluency: animal naming	18.2 (5.7)	18.7 (6.1)	17.5 (5.1)	17.6 (4.5)
B-MCCB, trial making test A	52.7 (30.2)	24.1 (2.5)	51.8 (26.0)	47.9 (25.2)

*B-MCCB* Brief MATRICS Consensus Cognitive Battery, *BPRS* Brief Psychiatric Rating Scale, *MANSA* Manchester Short Assessment of Quality of Life, *PSP* Personal and Social Performance Scale, *M* Mean, *sd* standard deviation

*p < 0.05; **p < 0.01; ***p < 0.001

Differences in the efficacy of the intervention at one year in the different diagnostic groups are reported in Table 5.

**Table 5:** Efficacy of the LIFESTYLE intervention on physical and mental health-related domains in the three diagnostic group

	Psychotic disorders		Bipolar disorders		Major depressive disorder	
	Baseline (*N* = 206)	One-year follow-up (*N* = 87)	Baseline (*N* = 195)	One-year follow-up (*N* = 86)	Baseline (*N* = 195)	One-year follow-up (*N* = 86)
*Physical health-related outcomes*						
BMI, kg/m^2^, M (sd)	32.4 (5.6)	31.0 (5.0)*	32.2 (4.7)	30.0 (4.7)**	33.1 (6.6)	31.9 (6.1)**
Body weight, M (sd)	93.3 (17.7)	89.3 (18.7)**	91.0 (16.3)	89.9 (13.3)*	86.3 (17.6)	84.9 (20.6)**
Waist circumference, M (sd)	111.9 (14.4)	109,2 (13.9)**	113.2 (14.8)	109.4 (11.9)***	106.8 (13.5)	103.4 (16.2)*
CIRS, severity index, M (sd)	0.2 (0.3)	0.2 (1.6)	0.3 (0.3)	.32 (.02)	0.4 (0.4)	0.3 (0.2)
CIRS, comorbidity index, M (sd)	0.4 (2.0)	.03 (.17)	0.1 (0.5)	.05 (.2)**	0.45 (1.6)	0.1 (0.2)
Systolic blood pressure, M (sd)	128.1 (14.0)	123.6 (14.2)	125.2 (12.7)	126.2 (9.5)	123.1 (13.7)	119.9 (13.7)
Diastolic blood pressure, M (sd)	82.0 (8.6)	81.7 (8.6)	80.9 (9.2)	80.4 (6.55)	79.0 (9.0)	75.9 (8.2)
Blood glucose, mg/dL, M (sd)	94.25 (20.6)	90.5 (29.6)	95.0 (28.3)	92.5 (20.4)	99.4 (34.0)	81.76 (17.0)**
Total cholesterolemia, mg/dL, M (sd)	184.6 (40.3)	173.8 (48.6)	190.5 (40.7)	190.6 (37.9)	194.8 (47.3)	184.8 (47.5)
Total LDL cholesterolemia, mg/dL, M (sd)	115.7 (33.3)	109.7 (42.7)	126.4 (79.0)	111.6 (33.1)*	120.3 (37.2)	119.2 (32.8)
Total HDL cholesterolemia, mg/dL, M (sd)	42.8 (13.4)	44.2 (15.4)	47.3 (16.1)	50.4 (14.5)**	47.7 (13.9)	65.1(50.9)***
Serum triglycerides, mg/dL, M (sd)	167.5 (92.2)	134.6 (93.5)**	171.4 (110.5)	136.45 (69.0)***	145.8 (80.1)	113.9 (48.8)***
Framingham risk score, total score, M (sd)	9.9 (4.9)	9.2 (4.5)	9.8 (4.6)	10.1 (3.7)	10.2 (4.0)	7.9 (4.2)
HOMA-IR-IR index, M (sd)	4.4 (4.2)	2.8 (3.5)*	4.8 (15.7)	3.7 (2.7)**	5.8 (9.1)	2.2 (0.8)**
Mental health-related outcomes						
BPRS affectivity subscale, M (sd)	8.8 (3.2)	7.3 (2.8)*	7.78 (2.5)	7.4 (2.4)*	10.1 (3.0)	6.4 (1.8)***
BRPS negative symptoms subscale, M (sd)	7.5 (2.9)	7.1 (2.7)*	7.2 (3.0)	7.0 (2.8)	8.9 (2.9)	6.7 (2.6)**
BPRS, positive symptoms subscale, M (sd)	5.7 (2.4)	5.5 (2.1)	5.1 (1.6)	5.2 (1.9)	4.8 (1.6)	4.5 (1.1)
BPRS, activity subscale, M (sd)	4.7 (1.8)	4.3 (1.2)*	4.7 (1.7)	4.2 (1.4)*	4.6 (2.4)	3.7 (1.1)*
BPRS, resistance subscale, M (sd)	4.9 (2.7)	4.8 (2.5)	3.5 (1.2)	3.7 (1.8)	3.3 (0.9)	3.1 (.3)
MANSA total score, M (sd)	3.8 (.09)	5.9 (0.9)**	4.2 (1.0)	5.3 (1.1)*	3.9 (0.9)	6.2 (1.3)**
PSP total score, M (sd)	66.4 (14.8)	66.9 (16.0)	69.9 (14.6)	68.0 (15.8)	68.9 (12.8)	73.7 (15.1)
B-MCCB, symbol coding	30.7 (11.7)	32.1 (12.8)	33.6 (15.0)	34.7 (13.5)	40.9 (13.1)	43.4 (16.2)
B-MCCB, category fluency: animal naming	15.5 (5.4)	17.6 (6.9)	18.6 (5.1)	18.4 (4.6)	21.2 (5.5)	21.6 (6.8)
B-MCCB, trial making test A	60.3 (38.7)	56.6 (24.9)	51.5 (26.9)	45.4 (23.1)	45.3(19.9)	3.6 (22.5)

*BMI* Body Mass Index, *B-MCCB* Brief MATRICS Consensus Cognitive Battery, *CIRS* Cumulative Illness Rating Scale, *HOMA-IR-IR* Homeostatic Model Assessment of Insulin Resistance, *LDL* Low-density lipoprotein, *HDL* High-density lipoprotein, *M* Mean, *sd* standard deviation

*p < 0.05; **p < 0.01; ***p < 0.001; Corrected p-value for multiple comparisons: ***p < 0.0002; **p < 0.002; *p < 0.01

### GEE models

Findings of the univariate analyses were confirmed at the GEE models. In fact, the LIFESTYLE intervention was associated with a reduction in BMI (B =  – 0.66, 95% CI  – 0.99 to  – 0.30, p < 0.001), body weight (B =  – 1.68, 95% CI  – 3.37 to 0.01, p < 0.05), waist circumference (B =  – 1.43, 95% CI  – 2.60 to  – 0.27, p < 0.05) and HOMA-IR-IR index (B =  – 1.54, 95% CI =  – 3.71 to 0.63, p < 0.05) (Table 6). Moreover, the GEE model confirmed that the LIFESTYLE intervention improves “Affectivity” at BPRS (B =  – 0.19; 95% CI  – 0.40 to 0.02, p < 0.05) and of quality of life (B = 1.6, 95% CI 0.00 to 2.31, p < 0.05) (Table 7).

**Table 6:** Generalized estimating equation models (GEE): efficacy of the LIFESTYLE intervention on physical health related outcomes

	BMI	Body weight	HOMA-IR-IR index	Waist circumference	CIRS Comorbidity index	Serum triglycerides	Total HDL Cholesterolemia
	B (95% CI)	B (95% CI)	B (95% CI)	B (95% CI)	B (95% CI)	B (95% CI)	B (95% CI)
Experimental treatment	– .66 (– .99 to – .30)***	– 1.68 (– 3.37 to .01)*	– 1.54 (– 3.71 to .63)*	– 1.43 (– 2.60 to – .27)*	– .04 (– .13 to .06)	7.33 (– 6.5 to 14.16)	– .20 (– 2.78 to – 3.18)
Gender, male	– .96 (– .1.70 to – .22)*	10.29 (8.36–12.22)***	.11 (– 1.36 to 1.58)	6.40 (4.29–8.52)****	– .09 (– .23 to .04)	43.23 (14.61–71.84)**	– 7.09 (– 8.70 to 5.49)***
Age	0.12 (.03 to – .05)	– .06 (– .25 to .14)	.03 (.00–.06)*	.13 (.00–.27)*	.01 (.00–.03)	.04 (– .51 to 1.35)	.09 (– .01 to .17)
Years of education	– .20 (– .34 to – .06)**	– .37 (– 1.11 to .37)	– .54 (– 1.16 to 0.09)	– .24 (– .75 to .27)**	.00 (– .02 to .02)**	1.83 (– .2.1 to 5.70)	– .20 (– .05 to 7.6)
Duration of illness, years	– .01 (– .03 to .02)	– .07 (– .12 to .09)	.00 (– .02 to .04)	.171 (.04 to .30)	.00 (.01–.00)	.01 (– .23 to .25)	.03 (– .03 to .02)
Symbol coding	– .01 (– .06 to .04)	– .05 (– .26 to .17)	.02 (– .09 to .14)	– .031 (– .15 to .09)	.00 (.00–.00)	– .53 (– 1.99 to .93)	– .04 (– .16 to .07)
Category fluency: animal naming	– .05 (– .16 to .07)	.05 (– 29 to .39)	.17 (0.15 to .47)	– .04 (– .09 to 0.01)	.00 (.00–.00)	.80 (– 1.30 to 2.91)	– .01 (– .04 to .02)
Trial making test A	– .01 (– .04 to -.00)	– .04 (– .12 to .03)	.00 (– 02 to .00)	– .04 (– .09 to .01)	.00 (.00–.00)	– .39 (– .62 to – .15)***	.00 (– .03 to .01)
PSP, total	– .91 (– 1.05 to – .01)	– .02 (– .02 to -.01)***	.00 (– .01 to .00)	– .013 (– .02 to .00)***	.01 (– .13 to .06)***	.04 (– .01 to .9)	.00 (.00–.00)

*BMI* Body Mass Index, *CIRS* Cumulative Illness Rating Scale, *HOMA-IR-IR* Homeostatic Model Assessment of Insulin Resistance, *B-MCCB* Brief MATRICS Consensus Cognitive Battery, *PSP* Personal and Social Performance, *BPRS* Brief Psychiatric Rating Scale. GEE have been adjusted for diagnosis and pharmacological treatments

*p < .05;**p < .01; ***p < .001

**Table 7:** Generalized estimating equation models (GEE): efficacy of the LIFESTYLE intervention on mental health-related outcomes

	BPRS subscales		MANSA total score	
	Affectivity subscale	Negative symptoms	Activity	
	B (95% CI)	B (95% CI)	B (95% CI)	B (95% CI)
Experimental treatment	– .19 (– .40 to .02)*	.24 (– .04 to .52)	.00 (– .19 to .18)	1.6 (.00–2.31)*
Gender, male	– .82 (– 1.60 to – .05)	– .77 (– 1.33 to – .20)**	.02 (– .19 to .22)	.22 (.02–.42)*
Age	.00 (– .04 to .05)	.03 (.00 to .06)	.00 (– .02 to .01)	.00 (– .01 to .00)
Years of education	– .12 (– .022 to .03)*	– .05 (– .16 to .07)	– .06 (– .11 to – .02)	.03 (– .02 to .00)
Duration of illness, years	.00 (– .02 to .02)	– .01 (– .03 to .01)	.00 (– .04 to .00)	– 8.52 (– .00 to .00)
B-MCCB Symbol coding	– .01 (– .025 to .02)	– .02 (– .04 to .01)	– .02 (– .04 to .07)	.00 (– .01 to .02)
B-MCCB Category fluency: animal naming	.03 (– .05 to .10)	.02 (– .06 to .10)	.02 (– .04 to .07)	.00 (– .04 to .02)
B-MCCB Trial making test A	.00 (– .02 to .07)	.13 (.00–.039	.00 (.00–.00)*	.00 (.00–.00)
PSP, total	– .01(– .01 to.01)	.00 (– .04 to .52)	.00 (– .19 to .18)	1.02 (.00–1.30)**

*B-MCCB* Brief MATRICS Consensus Cognitive Battery, *PSP* Personal and Social Performance, *BPRS* Brief Psychiatric Rating Scale. GEE have been adjusted for diagnosis and pharmacological treatment

* p < .05;**p < .01

Participants’ years of education and social functioning significantly influenced the efficacy of the intervention on BMI (B =  – 0.20, 95% CI =  – 0.34 to -0.06, p < 0.01) waist circumference (B = -0.24, 95%CI  – 0.75 to 0.27, p < 0.01) and BPRS “Affectivity” subscale (B =  – 0.12; 95% CI =  – 0.02 to 0.03, p < 0.05); in fact, higher scores at PSP were associated with reduced BMI (B =  – 0.91, 95% CI  – 1.05 to  – 0.01, p < 0.05), body weight (B =  – 0.02, 95% CI  – 0.02 to  – 0.01, p < 0.001), waist circumference (B =  – 0.01, 95% CI  – 0.02 to 0.00, p < 0.001) and improved quality of life (B = 1.02, 95% CI 0.00 to 1.30, p < 0.01). We also found that being male was associated with a smaller effect of the intervention on BMI (B = 0.96; 95% CI =  – 0.70 to  – 0.22, p < 0.05), body weight (B = 10.29; 95% CI 8.36 to 12.22, p < 0.001) and on waist circumference (B = 6.40; 95% CI 4.29 to 8.52).

## Discussion

Our findings confirm the main study hypothesis that the lifestyle behavioural intervention improves mental and physical health of patients with severe mental illness after one year. In fact, although the LIFESTYLE intervention was already effective at six months in reducing BMI, body weight and waist circumference [4], data on its long-term efficacy were missing. Moreover, at six-month the experimental intervention also contributed to promote healthy lifestyle behaviours, by increasing patients’ physical activity and healthy diet (i.e., reduced consumption of junk food and increased eating of fruit and vegetables) [32]. The 12-month findings presented in this paper confirm that the LIFESTYLE intervention is still effective at one year (B =  – 0.66,  – 1.68, and  – 1.43 for BMI, weight and waist circumference, respectively), also after controlling for several confounding factors. Moreover, at 12 months, the intervention significantly improved also the HOMA-IR index, which was not significant at six months. This finding is particularly interesting, since the importance of assessing cardiovascular risk scores (CVD) (i.e., HOMA-IR-IR or Framingham indexes) in patients with SMI has been recently highlighted [11, 15, 42, 43]. However, only in a few studies [44–46] considered CVD risk indexes among outcomes of behavioral interventions. Moreover, since changes in blood parameters are not easily detectable over a short period, the efficacy of psychosocial interventions on physical health should be evaluated in a longer period, such as the one used in our study.

Additionally, in the majority of available RCTs only one physical health parameter (i.e., body weight or BMI) was considered, while a complete assessment of anthropometric and haematological parameters was missing. We assessed both BMI and waist circumference as they are strongly correlated with cardiovascular [47] and metabolic syndromes [12] and considered more reliable parameters compared to body weight.

The magnitude of changes found in our study is substantially higher compared to changes reported by Bradley et al. in a recent metanalysis [8], in which the pooled positive effect of studies was in the range of 1.42 kg for weight loss (compared to 4.18 kg reported in our study), of 0.48 units for BMI (compared to 1.25 in our study), and of 0.87 cm for waist circumference (compared to 4.48 cm reported in our study). These differences can be due to several factors, one being the motivational approach included in each session of our experimental intervention. In fact, the motivational approach is one of the most effective strategies to promote behavioural changes and weight loss [48, 49]. Behavioural interventions including a motivational component are consistently associated with more evident benefits on patients’ physical health. Second, the development of a comprehensive approach to healthy living, which includes aspects related to the promotion of physical activity, the promotion of the Mediterranean diet principles, the provision of suggestions on how to quit smoking and to reduce risky behaviours, the focus on the regularization of circadian rhythms and on the improvement of adherence to medical advice (including medications). A third aspect that may have contributed to foster the effects of the LIFESTYLE intervention is the group format, in which patients with different diagnoses discussed together aspects not directly related to their mental illness, but rather to their common daily life difficulties, which is in line with the transdiagnostic paradigm of mental health care [50].

Patients’ socio-demographic and clinical characteristics, such as gender, educational level, and psychosocial functioning significantly influenced the impact of the LIFESTYLE intervention on physical health-related domains. In particular, gender differences in BMI, body weight and waist circumference indicate that the LIFESTYLE intervention was less effective in men compared to women, suggesting the need for a personalized approach to patients with severe mental illnesses and for different approaches to men and women. It may be that male patients, being less motivated to body weight changes than females, tend to perform less frequently physical activity or to follow healthy eating suggestions, with a consequent reduced efficacy of the intervention [51]. In clinical practice, the presence of a gender difference should be considered while planning supportive interventions, with the need to find alternative strategies for BMI reduction when male patients are involved. However, the gender effect on the efficacy of psychosocial interventions on physical health domains is still yet to be fully elucidated and further analyses will be performed from our dataset.

We also found that the level of education has a significant impact on the efficacy of the LIFESTYLE approach, especially on BMI and waist circumference. It may be that less educated patients experience more difficulties in understanding content of sessions and the importance of implementing healthier habits. Thus, information should be adapted in content and language to the level of patients’ education in order to increase intervention effectiveness. Also patient’s psychosocial functioning significantly influenced the efficacy of the intervention. In fact, patients with a poor psychosocial functioning reported less benefits from the intervention on BMI, body weight and waist circumference. It may be that these patients are less motivated to participate in social and group activities, suggesting the need for a multilevel intervention, aimed at improving patients’ psychosocial functioning first and focusing on physical health only later [52–57].

The LIFESTYLE intervention was also significantly effective on several mental health domains. In fact, according to the GEE models, the experimental intervention reduced patients’ anxiety and depressive symptoms and improved their quality of life (QoL). The improvement of patients’ QoL is crucial to strengthen their motivation toward healthy lifestyle behaviours and thus it should always be the target of behavioural interventions focusing on patients’ physical health [58]. This result is in line with those reported by Sampasa-Kanyinga et al. [59] and Kleppang et al. [60], which reported that the improvement of healthy lifestyle is associated with reduced anxiety and depressive symptoms as well as suicide ideation and attempts. This result might be explained by the inclusion in the experimental intervention of a structured programme to promote physical exercise, which is associated with brain plasticity and neurogenesis that, in turn, increase patients’ cognitive performance, problem-solving ability and skills to cope with environmental stressors [61]. The reduction of anxiety symptoms is also particularly relevant, since these symptoms in patients with severe mental disorders are frequently associated with the adoption of unhealthy lifestyle behaviours, in particular heavy smoking, sedentary habits, emotional eating and substance abuse [62]. Moreover, the presence of depressive symptoms may reduce patients’ motivation to attend physical health consultations [63], adherence to pharmacological treatments and to behavioural changes [61, 64]. Thus, the improvement of depressive symptoms further strengths the importance to provide behavioural interventions, such as ours, to patients with severe mental disorders in routine care.

The following limitations shall be mentioned. First, the high drop-out rate which is, however, in line with other trials carried out on other psychosocial interventions for people with severe mental illnesses [33, 65], where drop-out rates of 40% were reported. Despite this, the final sample size at one year can be still considered satisfactory compared with previous studies. Several strategies could be implemented in order to increase the rate of participants who complete psychoeducational or behavioural interventions, such as the use of electronic reminders (e.g., phone calls, emails, instant messages), the availability of dedicated staff members and of rooms/spaces to run the intervention [33, 66]. Future implementation strategies could include the use of web-based components, such as smartphone apps and wearable devices, for increasing real-time interaction with participants [67]. Another limitation is the fact that the role of psychiatric medications on patients’ physical parameters was not assessed. However, we tried to overcome this bias by controlling the GEE for pharmacological treatments, and by including in the study only patients in a stable phase of the illness. Another possible limitation is the shorter duration of the intervention provided to the control group. However, most RCTs on psychosocial interventions explore the efficacy of the experimental interventions against treatment as usual, or against a waiting list (meaning no active comparator) or against no intervention. Thus, we considered the comparison between two active interventions an added value of our protocol.

In conclusion, the LIFESTYLE experimental intervention improved both physical and mental health domains at a 12-month follow-up in a sample of overweight patients with severe mental disorders. This supports the notion that appropriate support can help patients to improve their lifestyle behaviours and to achieve a healthy living. If we really want to reduce the mortality gap in patients with severe mental disorders, more efforts need to be done in order to increase the availability of these interventions in routine clinical settings [68], eventually also through the use of digital means [69, 70].

## Data Availability

Data published in this paper are available at the corresponding author upon request.
